# Prevalence and Distribution of Ossified Lesions in the Whole Spine of Patients with Cervical Ossification of the Posterior Longitudinal Ligament A Multicenter Study (JOSL CT study)

**DOI:** 10.1371/journal.pone.0160117

**Published:** 2016-08-22

**Authors:** Takashi Hirai, Toshitaka Yoshii, Akio Iwanami, Kazuhiro Takeuchi, Kanji Mori, Tsuyoshi Yamada, Kanichiro Wada, Masao Koda, Yukihiro Matsuyama, Katsushi Takeshita, Masahiko Abematsu, Hirotaka Haro, Masahiko Watanabe, Kei Watanabe, Hiroshi Ozawa, Haruo Kanno, Shiro Imagama, Shunsuke Fujibayashi, Masashi Yamazaki, Morio Matsumoto, Masaya Nakamura, Atsushi Okawa, Yoshiharu Kawaguchi

**Affiliations:** 1 Department of Orthopedic Surgery, Tokyo Medical and Dental University, 1-5-45 Yushima, Bunkyo Ward, Tokyo, 113–8519, Japan; 2 Department of Orthopedic Surgery, School of Medicine, Keio University, 35 Shinanomachi, Shinjuku Ward, Tokyo, 160–8582, Japan; 3 Department of Orthopedic Surgery, National Hospital Organization Okayama Medical Center, 1711–1 Tamasu, Okayama, Okayama, 701–1154, Japan; 4 Department of Orthopedic Surgery, Shiga University of Medical Science, Tsukinowa-cho, Seta, Otsu, Shiga, 520–2192, Japan; 5 Department of Orthopedic Surgery, Hirosaki University Graduate School of Medicine, 53 Honcho, Hirosaki, Aomori, 036–8203, Japan; 6 Department of Orthopedic Surgery, Chiba University Graduate School of Medicine, 1-8-1 Inohana, Chuo Ward, Chiba, Chiba, 260–0856, Japan; 7 Department of Orthopedic Surgery, Hamamatsu University School of Medicine, 1-20-1 Handayama, Hamamatsu, Shizuoka, 431–3125, Japan; 8 Department of Orthopedics, Jichi Medical University, 3311–1 Yakushiji, Shimotsuke, Tochigi, 329–0498, Japan; 9 Department of Orthopedic Surgery, Graduate School of Medicine and Dental Science, Kagoshima University, 8-35-1 Sakuragaoka, Kagoshima, Kagoshima, 890–8520, Japan; 10 Department of Orthopedic Surgery, University of Yamanashi, 1110 Shimokato, Chuo Ward, Yamanashi, 409–3898, Japan; 11 Department of Orthopedic Surgery, Surgical Science, Tokai University School of Medicine, 143 Shimokasuya, Isehara, Kanagawa, 259–1143, Japan; 12 Department of Orthopedic Surgery, Niigata University Medicine and Dental General Hospital, 1–754 Asahimachidori, Chuo Ward, Niigata, Niigata, 951–8520, Japan; 13 Department of Orthopaedic Surgery, Tohoku Medical and Pharmaceutical University, 1-12-1 Fukumuro Miyaginoku, Sendai, 983–8512, Japan; 14 Department of Orthopaedic Surgery, Tohoku University School of Medicine, 1–1 Seiryomachi, Aoba Ward, Sendai, Miyagi, 980–8574, Japan; 15 Department of Orthopedic Surgery, Nagoya University Graduate School of Medicine, 65 Tsurumaicho, Showa Ward, Nagoya, Aichi, 466–0065, Japan; 16 Department of Orthopedic Surgery, Graduate School of Medicine, Kyoto University, 54 Kawaharacho, Shogoin, Sakyo Ward, Kyoto, Kyoto, 606–8507, Japan; 17 Department of Orthopedic Surgery, Faculty of Medicine, University of Tsukuba, 2-1-1 Amakubo, Tsukuba, Ibaraki, 305–8576, Japan; 18 Department of Orthopedic Surgery, Faculty of Medicine, University of Toyama, 2630 Sugitani, Toyama, Toyama, 930–0194, Japan; 19 Working group of the CT study, Japanese Multicenter Research Organization for Ossification of the Spinal Ligament (JOSL), Tokyo, Japan; Mayo Clinic Minnesota, UNITED STATES

## Abstract

Ossification of the posterior longitudinal ligament (OPLL) can cause severe and irreversible paralysis in not only the cervical spine but also the thoracolumbar spine. To date, however, the prevalence and distribution of OPLL in the whole spine has not been precisely evaluated in patients with cervical OPLL. Therefore, we conducted a multi-center study to comprehensively evaluate the prevalence and distribution of OPLL using multi-detector computed tomography (CT) images in the whole spine and to analyze what factors predict the presence of ossified lesions in the thoracolumbar spine in patients who were diagnosed with cervical OPLL by plain X-ray. Three hundred and twenty-two patients with a diagnosis of cervical OPLL underwent CT imaging of the whole spine. The sum of the levels in which OPLL was present in the whole spine was defined as the OP-index and used to evaluate the extent of ossification. The distribution of OPLL in the whole spine was compared between male and female subjects. In addition, a multiple regression model was used to ascertain related factors that affected the OP-index. Among patients with cervical OPLL, women tended to have more ossified lesions in the thoracolumbar spine than did men. A multiple regression model revealed that the OP-index was significantly correlated with the cervical OP-index, sex (female), and body mass index. Furthermore, the prevalence of thoracolumbar OPLL in patients with a cervical OP-index ≥ 10 was 7.8 times greater than that in patients with a cervical OP-index ≤ 5. The results of this study reveal that the extent of OPLL in the whole spine is significantly associated with the extent of cervical OPLL, female sex, and obesity.

## Introduction

Since first being documented in 1838 [1], ossification of the posterior longitudinal ligament (OPLL) has been recognized as a heterotopic bone formation in the posterior longitudinal ligament of the spine. Although OPLL is considered a rare disease in Western countries [2], it is a major disorder in Asia and often causes severe myelopathy or spinal cord injury. The prevalence of OPLL in Japanese populations has been reported to range from 1.9% to 4.3% [3], compared with only 0.1% to 1.3% in the United States [4–6]. Therefore, close attention has been paid to genetic factors that might be associated with the development of OPLL, such as mutations, single-nucleotide polymorphisms, and haplotypes [7–13].

Various studies have documented the incidence of cervical OPLL. However, it is difficult to precisely evaluate OPLL in the thoracic spine on plain radiographs, especially at the upper and middle levels, because the shoulders obscure the findings of the upper thoracic spine. Recently, increased use of multi-detector computed tomography (CT) has contributed to recognition of a higher prevalence of ossified lesions than has been found by the previous method using plain X-rays [14]. A previous single-institution CT study demonstrated that patients with cervical OPLL had a high incidence of OPLL in the thoracolumbar spine [9]. It has been well documented that prognosis and surgical outcomes are worse in patients with multiple-regional OPLL than in patients with cervical lesions alone [15]. Because thoracolumbar OPLL often coexists with cervical OPLL and sometimes causes severe and irreversible paralysis [16], it is clinically important to recognize thoracolumbar OPLL and clarify the factors associated with the extent of OPLL in the whole spine.

Although a single-institution CT study has reported the prevalence of ossified lesions in patients with cervical OPLL [9], we conducted the present multicenter CT study with the aim of not only evaluating the prevalence of OPLL in the whole spine among patients with cervical OPLL in order to strengthen the evidence, but also using a multiple regression model to investigate the factors that correlate with the presence and distribution of ossified lesions in the whole spine.

## Materials and Methods

### Patients and methods

Prior to enrollment in this study, patients at each participating institution provided written informed consent, and the study was approved by the institutional review board of the University of Toyama. We undertook this study to utilize data from the Japanese Multicenter Research Organization for Ossification of the Spinal Ligament instituted by Japanese Ministry of Health, Labour and Welfare. This study included patients with OPLL in the cervical spine diagnosed based on findings of plain neck radiographs who had symptoms such as neck pain, numbness in the upper or lower extremities, clumsiness, or gait disturbance. Each patient underwent CT imaging of the whole spine at one of the 20 institutions to which the members of the research group belonged. Patients who had undergone anterior decompression surgery for the treatment of OPLL or who were younger than 15 years old were excluded. A total of 322 cases were collected, including 242 men and 80 women, all Japanese Asian, with an average age of 64.6 years (range, 30–93 years).

### Evaluations

**Fig 1:**
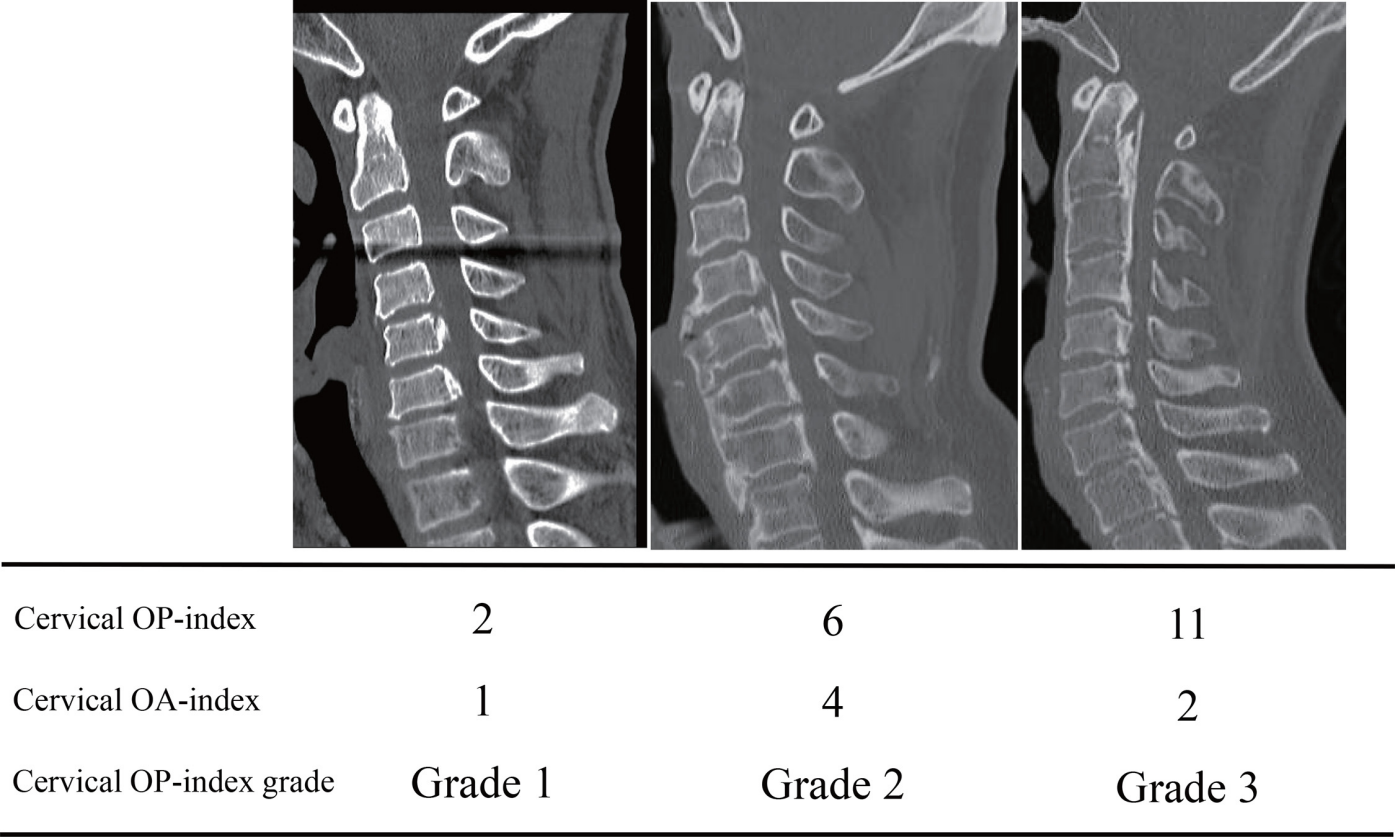
Cervical ossification of the posterior longitudinal ligament index (OP-index) and ossification of the anterior longitudinal ligament index (OA-index) are shown. Cervical OP-index classification was defined according to the cervical OP-index (Grade 1, cervical OP-index ≤ 5; Grade 2, cervical OP-index 6–9; and Grade 3, cervical OP-index ≥ 10).

Age, sex, presence of diabetes, and body mass index (BMI) were collected as basic clinical data (S1 Table). CT images of the whole spine, including the cervical, thoracic, and lumbosacral spine from the occipital bone to the sacrum, were obtained in each patient. The incidence of OPLL in the cervical spine from the clivus to C7 and in other spinal regions from T1 to S1 was evaluated on mid-sagittal CT images. Image analysis was independently performed by five senior spine surgeons (T.H., K.T., K.M., A.I., and T.Y.). To quantify hyperostosis of the posterior longitudinal ligament, the distribution of OPLL at each vertebral body and intervertebral disc level was recorded, and the number of levels at which OPLL was present was defined as the ossification index (OP-index), as described previously [9]. The number of ossified lesions in the cervical spine was defined as the cervical OP-index. Patients were categorized into three groups according to the cervical OP-index: Grade 1, cervical OP-index ≤ 5; Grade 2, cervical OP-index 6–9; and Grade 3, cervical OP-index ≥ 10 (cervical OP-index classification). In addition to the OP-index, the sum of the intervertebral segments showing ossification of the anterior longitudinal ligament (OALL; cervical OA-index) was noted (S1 Table). The cervical OP-index classification and two indexes are shown in Fig 1.

Prior to image review, all testers read images from the same 20 patients to check inter-observer agreement. The average Kappa coefficient of inter-observer agreement was 0.76 (95% confidence interval [CI] = 0.71–0.81). Kappa values 0.00–0.20 were considered to indicate slight agreement; 0.21–0.40, fair agreement; 0.41–0.60, moderate agreement; 0.61–0.80, substantial agreement; and 0.811.00, almost perfect agreement [17]. Therefore, this finding indicates substantial agreement and consistency with the results of the previous study [9]. We also evaluated the degree of OPLL occupying the cervical spinal canal, with classification of the canal narrowing ratio (CNR) [18] at the most compressed segment defined as follows: Grade 1, 0% < CNR ≤ 25%; Grade 2, 25% < CNR ≤ 50%; Grade 3, 50% < CNR ≤ 75%; and Grade 4, CNR > 75%. First, we compared male and female populations in terms of the physical and radiologic data. We next evaluated the usefulness of the cervical OP-index classification for predicting the presence of OPLL in the thoracolumbar spine. Finally, we used a multiple regression model to investigate the factors associated with the OP-index in all patients.

### Statistical analysis

Student’s unpaired *t* test was used to analyze differences in age, BMI, cervical OA- and OP-indexes, and OP-index of the whole spine between male and female participants. The χ^2^ test was used to analyze differences in sex and the presence of diabetes. Tukey’s post hoc test was applied to compare the three cervical OP-index classification groups. A logistic regression model was conducted to investigate whether cervical OP-index classification predicted the presence of OPLL in the thoracolumbar spine and the incidence of patients with an OP-index ≥ 20. Additionally, to identify factors that significantly influence the OP-index, predictive factor analysis was performed by multiple regression analysis with a forward stepwise procedure using SPSS 22.0 for Windows (SPSS Institute, Chicago, IL).

All data are expressed as means ± standard deviation (SD). A *p* value less than 0.05 was considered to indicate a statistically significant difference.

## Results

### Prevalence of OPLL in the thoracolumbar spine in patients with cervical OPLL

One hundred eighty-one patients (56.2%) had OPLL in multiple spinal regions. We compared patients who had OPLL only in the cervical spine with those who had OPLL in multiple regions (Table 1). No significant differences were found in terms of age, comorbidity of diabetes, BMI, or distribution of CNR categories. However, more women than men had coexisting OPLL in the thoracolumbar spine. The cervical OP-index, OA-index, and total OP-index were significantly greater in patients with OPLL in multiple spinal regions than in those with OPLL in the cervical spine alone (Table 1).

**Table 1:** Demographics of patients with cervical OPLL only and patients with OPLL in multiple spinal regions.

	Cervical only (n = 141)	Multiple spinal regions (n = 181)	*p*
Age (years)	65.4 ± 10.8	64.2 ± 11.5	0.32
Sex (M/F)	118/23	124/57	< 0.01
Diabetes mellitus (%)	30.5%	32.6%	0.69
BMI	25.3 ± 4.4	26.1 ± 5.1	0.10
CNR category			
Grade 1 (0% < CNR ≤ 25%)	26.2%	19.3%	-
Grade 2 (25% < CNR ≤ 50%)	46.8%	46.5%	-
Grade 3 (50% < CNR ≤ 75%)	22.0%	29.8%	-
Grade 4 (CNR > 75%)	5.0%	4.4%	-
Cervical OP-index	4.86 ± 2.5	6.59 ± 3.0	< 0.01
OA-index	1.69 ± 1.7	2.24 ± 1.8	< 0.01
OP-index	4.86 ± 2.5	12.6 ± 7.0	< 0.01

Data are expressed as means ± standard deviations where appropriate. BMI, body mass index; CNR, canal narrowing ratio; OP-index, ossification index of OPLL; OA-index, ossification index of anterior longitudinal ligament of the cervical spine.

### Difference in the distribution of ossified lesions between male and female subjects

Demographic data are shown in Table 2. Age, comorbidity of diabetes, BMI, distribution of CNR categories, and the cervical OP-index were similar between male and female participants. The percentage of patients who had OPLL in the thoracolumbar spine was significantly greater among women than among men. The average cervical OA-index was significantly higher in men than in women; interestingly, however, the OP-index of the whole spine was significantly greater in women.

**Table 2:** Demographics of male and female patients.

	Male (n = 242)	Female (n = 80)	*p*
Age (years)	64.7 ± 11.6	64.6 ± 10.0	0.90
Coexistence of OPLL in thoracolumbar spine (%)	51.2%	71.3%	< 0.01
Diabetes mellitus (%)	31.8%	31.3%	0.92
BMI	25.8 ± 4.8	25.5 ± 4.7	0.62
CNR category			
Grade 1 (0% < CNR ≤ 25%)	21.1%	26.3%	-
Grade 2 (25% < CNR ≤ 50%)	45.0%	48.8%	-
Grade 3 (50% < CNR ≤ 75%)	29.8%	18.7%	-
Grade 4 (CNR > 75%)	4.1%	6.2%	-
Cervical OP-index	5.86 ± 2.9	5.75 ± 3.0	0.78
OA-index	2.20 ± 1.7	1.4 ± 1.7	< 0.01
OP-index	8.24 ± 5.5	12.1 ± 9.0	< 0.01

Data are expressed as means ± standard deviations where appropriate. BMI, body mass index; CNR, canal narrowing ratio; OP-index, ossification index of OPLL; OA-index, ossification index of anterior longitudinal ligament of the cervical spine.

**Fig 2:**
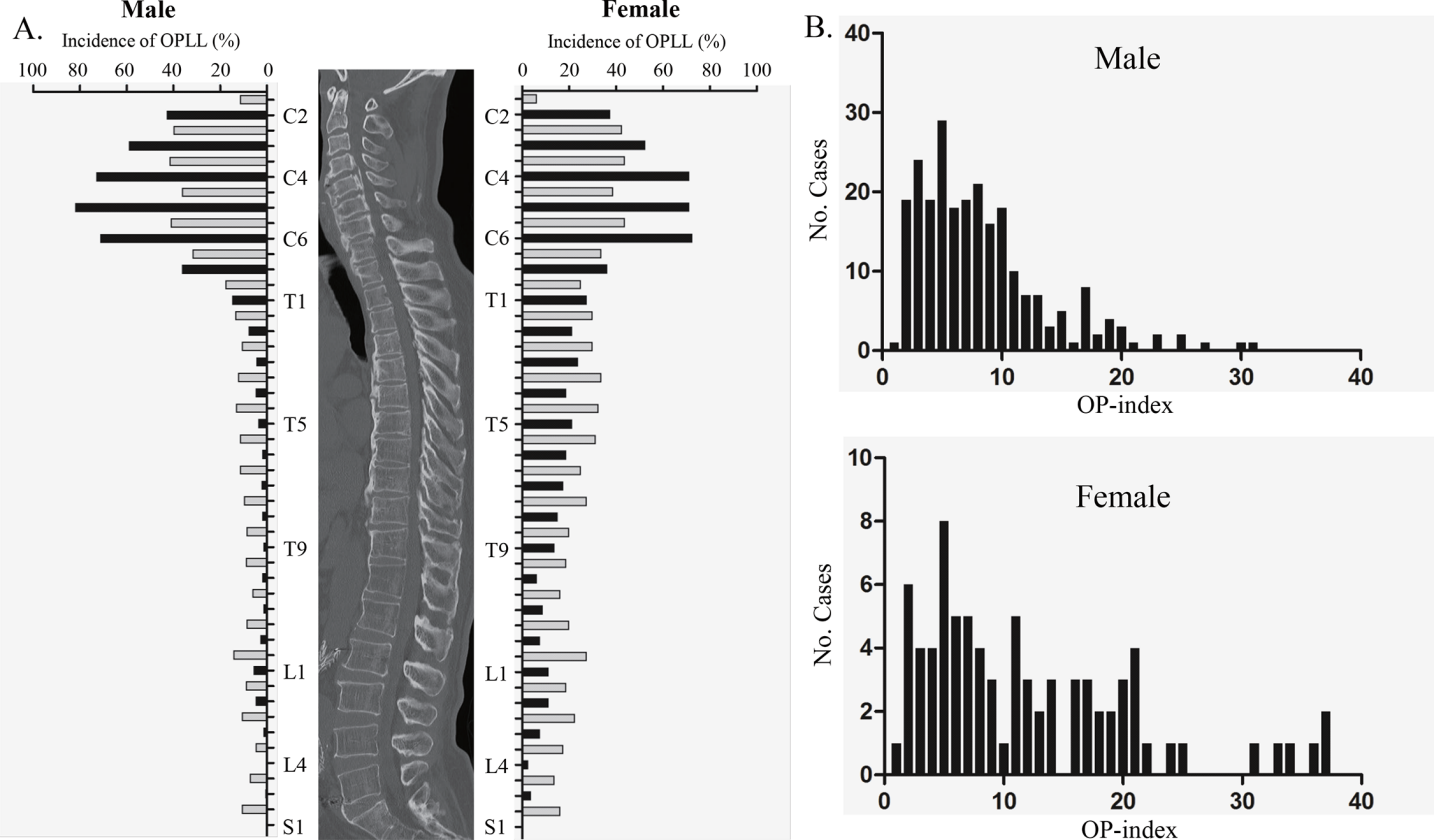
A: Incidence of ossification of the posterior longitudinal ligament (OPLL) at each vertebral and intervertebral level (black bars, vertebral levels; gray bars, intervertebral levels). B: Histograms of the OP-index of the whole spine for male and female patients.

The distribution of ossified lesions in the whole spine is shown in Fig 2A. Overall, female patients were more likely than male patients to have OPLL broadly from the cervical to the lumbar spine. Ossified lesions at the vertebral level of the middle cervical spine were confirmed frequently, regardless of sex. The highest peak of OPLL incidence along the thoracic spine was found at the intervertebral segment of T3/4 in female but not male patients. Similarly to the thoracic spine, ossified lesions of the lumbar spine likely exist at intervertebral rather than vertebral levels.

We next compared histograms of the OP-index of the whole spine for male and female patients (Fig 2B). A flat waveform from a low to high OP-index could be observed in female patients, whereas a single peak was found in male patients. The proportion of patients with a high OP-index (≥ 20) was significantly higher among women (20%) than among men (4.5%).

**Fig 3:**
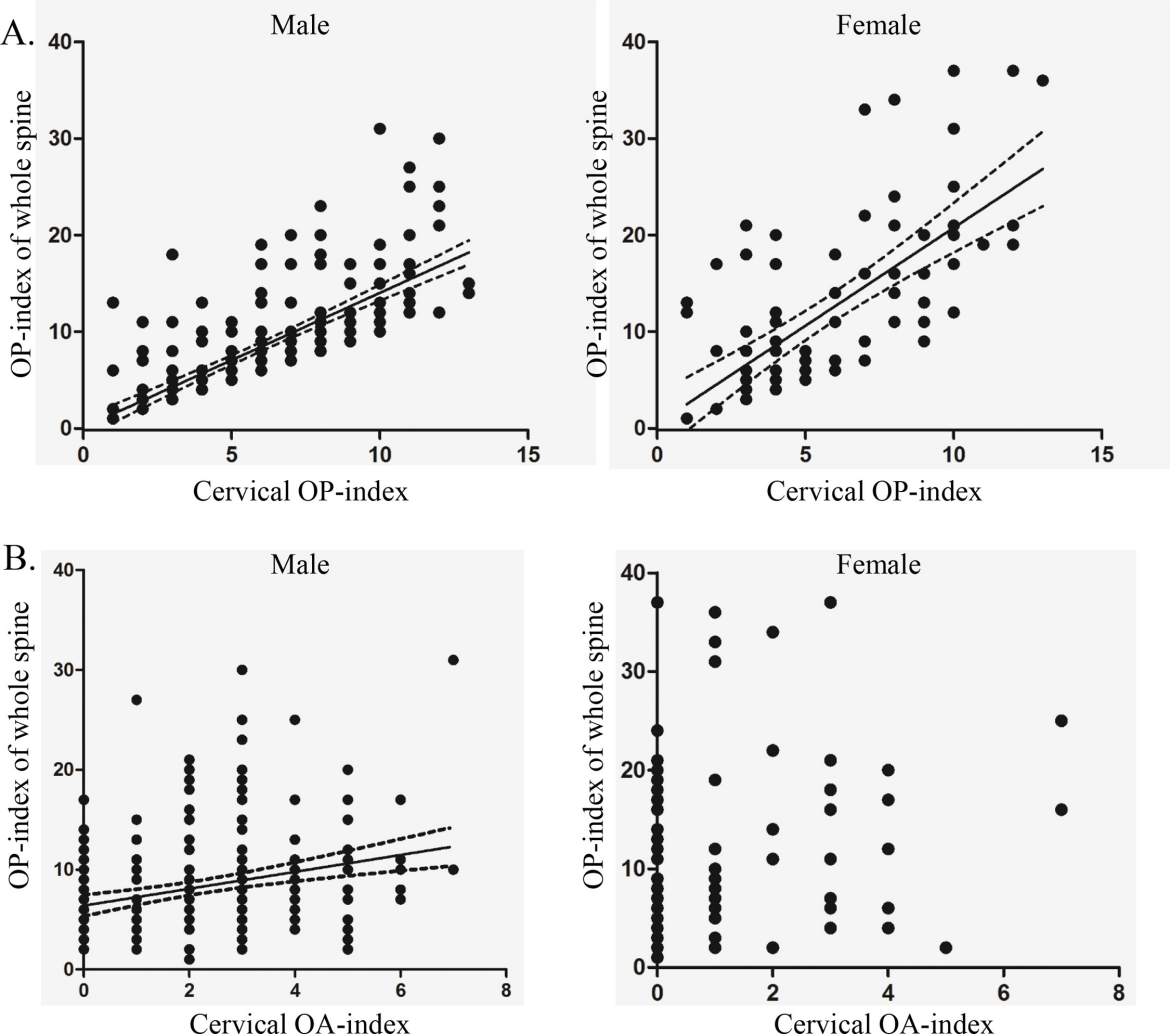
A: The OP-index correlates significantly with the cervical OP-index in both men (*p* < 0.001, R^2^ = 0.536) and women (*p* < 0.001, R^2^ = 0.464). B: The OP-index of the whole spine was significantly associated with the cervical OA-index in men (*p* < 0.001, R^2^ = 0.0735), but not in women.

We also investigated the associations between the OP-index of the whole spine and either the cervical OP-index or the cervical OA-index. In male patients, the OP-index correlated significantly with both the cervical OP-index and cervical OA-index (Fig 3A and 3B). In female patients, however, only the OP-index was significantly associated with the cervical OP-index (Fig 3A and 3B).

### The cervical OP-index classification for prediction of the presence of OPLL in the thoracolumbar spine

**Fig 4:**
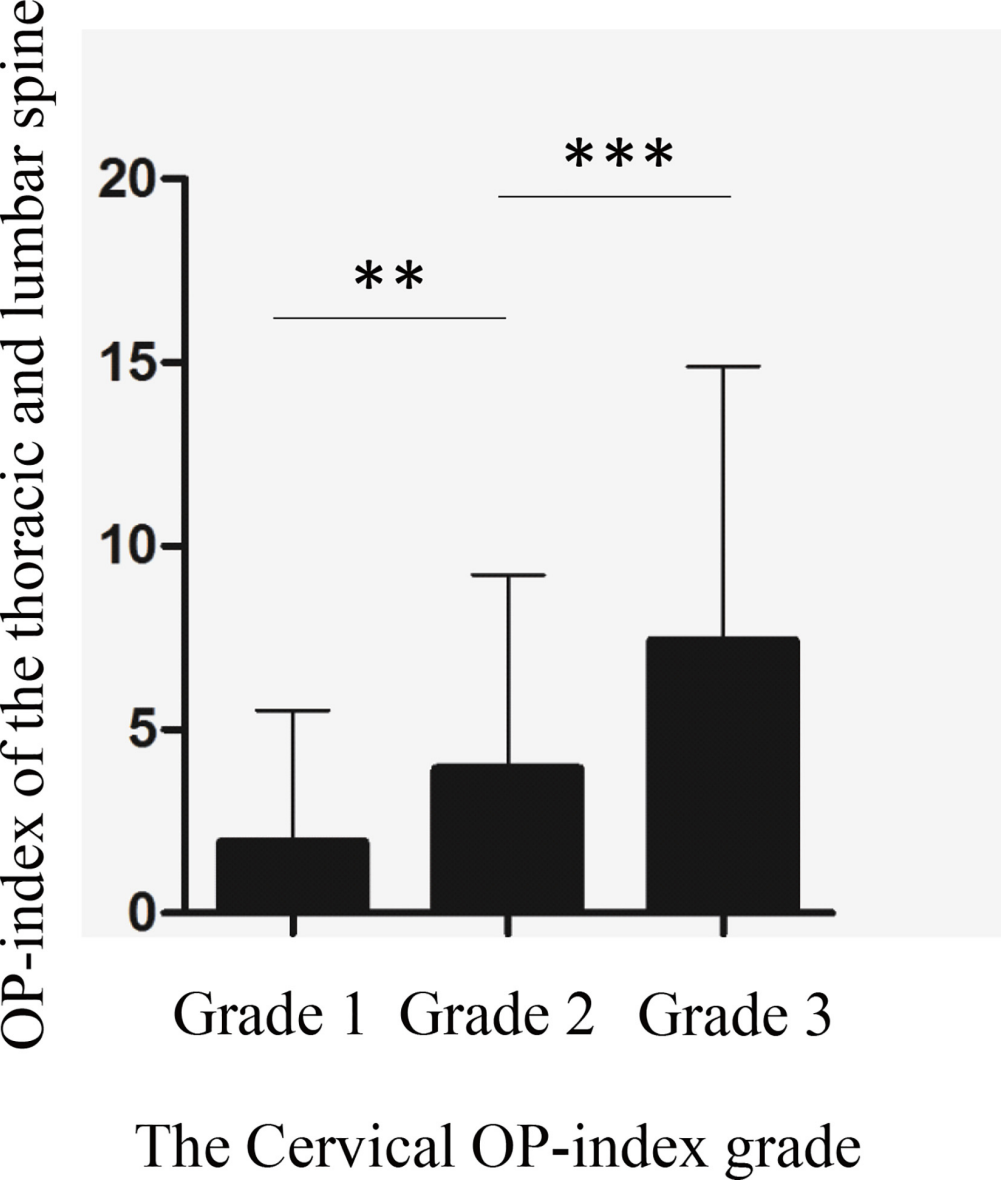
Patients were categorized into three groups according to the cervical OP-index: Grade 1, cervical OP-index ≤ 5; Grade 2, cervical OP-index 6–9; and Grade 3, cervical OP-index ≥ 10. The number of ossified lesions in the thoracolumbar spine was significantly different among the three grades (**p < 0.01 and ***p < 0.001).

We categorized patients into three grades according to the cervical OP-index (Table 3). The mean OP-index of the thoracic and lumbar spine was 1.93 for Grade 1, 3.93 for Grade 2, and 7.37 for Grade 3 (*p* < 0.001), indicating that there were significant differences in the number of ossified lesions in the thoracolumbar spine among the three cervical OP-index grades (Fig 4). We found that the number of levels with OPLL and the incidence of OPLL in spinal regions other than the cervical spine increased with increasing grade (Table 3). A logistic regression model showed significant correlation between the cervical OP-index grade and the incidence of OPLL in the thoracolumbar spine (hazard ratio 2.795, 95% CI = 1.946–4.015, *p* < 0.001; Table 4), indicating that the prevalence of thoracolumbar OPLL in patients with a cervical OP-index ≥ 10 was 7.8-fold that in patients with a cervical OP-index ≤ 5. We also found that the grading system could predict the incidence of patients with an OP-index ≥ 20 (hazard ratio 6.360, 95% CI = 3.352–12.070, *p* < 0.001; Table 4).

**Table 3:** Categorization of the cervical OP-index and relationship between the classification and incidence of ossified lesions in the lumbar and thoracic spine.

Cervical OP-index grade	No. of cases	Presence of OPLL in thoracic or lumbar spine	Proportion of patients with OP-index ≥ 20
Grade 1 (1 ≤ cervical OP-index ≤ 5)	168	42.3%	1.2%
Grade 2 (6 ≤ cervical OP-index ≤ 9)	108	70.4%	8.3%
Grade 3 (cervical OP-index ≥ 10)	46	82.6%	32.6%

OPLL, ossification of the posterior longitudinal ligament; OP-index, ossification index of OPLL.

**Table 4:** Increased risk of OPLL in multiple regions based on the cervical OP-index grade.

Characteristic	Increase in cervical OP-index grade	HR for incidence of OPLL in multiple regions	95% CI	*p*
Presence of OPLL in thoracolumbar spine	+1 Grade	2.795	1.946–4.015	< 0.001
OP-index ≥ 20	+1 Grade	6.360	3.352–2.070	< 0.001

HR, hazard ratio; OPLL, ossification of the posterior longitudinal ligament; OP-index, ossification index of OPLL; CI, confidence interval.

### Multiple regression model analyzing factors correlated with the OP-index of the whole spine

To identify the factors that influence the OP-index of the whole spine, a stepwise multiple regression analysis was conducted with independent variables such as age, sex, presence of diabetes, BMI, CNR category, cervical OP-index, and cervical OA-index. Factors influencing the OP-index included the cervical OP-index, sex, and BMI (Y = -8.707 + 4.108X_1_ + 1.558X_2_ + 0.143X_3_; Y, OP-index of the whole spine; X_1_, sex [male = 0, female = 1]; X_2_, cervical OP-index; X_3_, BMI [kg/m^2^]; Table 5).

**Table 5:** Factors influencing the OP-index of the whole spine.

Factor	Standardized β	t	*p*	Adjusted R^2^	F	*p*
Cervical OP-index	0.673	17.524	< 0.001	0.529	120.6	< 0.001
Sex (0, Male; 1, Female)	0.264	6.881	< 0.001			
BMI (kg/m^2^)	0.107	2.774	0.006			

OP-index, ossification index of OPLL; BMI, body mass index.

## Discussion

Epidemiological studies have shown that genetic background is a contributory factor in OPLL [8,19]. The current study revealed that 56.2% of patients with cervical OPLL had coexisting ossified lesions in the thoracolumbar spine. The incidence of OPLL in the thoracolumbar spine is much higher in this group compared with the general population [14]. This finding suggests that patients with cervical OPLL generally have a predisposition to hyperostosis in the posterior longitudinal ligament in the whole spine. It is known that cervical OPLL has a male predominance of 2:1 to 3:1 [20]. On the other hand, thoracic OPLL has a female predominance of approximately 3:1 [14]. Our current study showed that patients with a high OP-index of the whole spine were predominantly women. This finding is consistent with evidence that female patients tend to have OPLL in the thoracic spine—the area of the spine in which the number of vertebral and intervertebral levels is greatest—and indicates that hormonal factors could affect the development of OPLL in the whole spine. Although the details of why patients with multiple ossified lesions are predominantly female remain unclear, several studies have suggested possible mechanisms linking sex and hyperostosis [9,21–23]. Serum estrogen level has been shown to correlate with the onset and extension of ossified lesions, and estrogen is thought to stimulate osteoblast-like cells with the aid of trophic factors [9,21,22]. A TGF-beta 1 polymorphism has been found to be partially related to the area of ossified lesions via stimulation of estrogen [23]. Ikeda et al. showed that serum leptin and insulin concentrations were significantly increased in female patients with OPLL, and serum leptin level correlated positively with not only the number of vertebral levels with OPLL, but also the extension of ossified lesions in the thoracic and/or lumbar spine [24]. These metabolism-related factors could affect the onset and development of OPLL in the whole spine as well as the difference in the prevalence of OPLL between men and women.

OALL has not been widely discussed in terms of etiology or association with OPLL. Since Resnick et al. [25] coined the term “diffuse idiopathic skeletal hyperostosis” (DISH) for Forestier’s disease and recognized ossification of spinal ligaments as a subset of this entity, several reports have shown that the coexistence of OPLL and DISH is relatively common. Although both anterior and posterior longitudinal ligaments run longitudinally along the vertebral body from the cervical spine to the sacrum, the pathogenesis of these states remains unclear. A recent multicenter study [26] demonstrated that OPLL detected on radiographs was significantly associated with the presence of DISH in the Japanese population in men but not in women. Similarly, the current study revealed that the cervical OA-index was significantly related to the OP-index of the whole spine only in male patients. Thus, we speculate that the mechanism of onset and development of ossified lesions in ALL or PLL could differ between men and women.

A previous study [27] investigated the distribution and localization of cervical and thoracic OPLL using plain X-rays; however, it is often difficult to perform a detailed evaluation of thoracic OPLL using this method. Ossification in the thoracic spine occasionally has a critical influence on the spinal cord and leads to severe myelopathy [28]; hence, more precise evaluation of not only cervical lesions but also thoracolumbar lesions should be performed for patients with OPLL. Fujimori et al. reported that the prevalence of thoracic OPLL was 0.7% among 1500 Japanese patients who underwent positron emission tomography and computed tomography, and more than half of the patients with thoracic OPLL also had cervical OPLL [29]. Therefore, it is reasonable to identify unrecognized thoracolumbar lesions, which are undetectable on screening radiographs, in patients with cervical OPLL using multi-detector CT. Previously, Kawaguchi et al. investigated the distribution of OPLL using multi-detector CT in patients with cervical OPLL. This single-institutional study demonstrated that patients with cervical OPLL also had a high incidence of OPLL in the thoracolumbar spine [9]. To increase the generalizability of this finding and strengthen the evidence, we conducted a multicenter study with a wider population base covering various geographic locations. In addition, we extensively analyzed CT data with a large sample size to identify potential prognostic factors for the presence of severe ossified lesions in the whole spine of patients with OPLL in the cervical spine. The results of the multiple regression analysis revealed that the number of ossified lesions in the whole spine was significantly correlated with female sex, obesity, and the number of cervical ossified lesions. Notably, because there was a strong correlation between the number of cervical ossified lesions and the OP-index of the whole spine, we defined a new cervical OP classification and found that the incidence of severe OPLL (OP-index of ≥ 20) was 1.2% in Grade 1, 6.3% in Grade 2, and 32.6% in Grade 3 (Table 3). In addition, logistic regression analysis revealed a significant correlation between the grading and the incidence of multiple ossified lesions in the whole spine. We therefore recommend that patients with a high cervical OP-index (ie, Grade 3), especially those who are obese and female, should consider undergoing CT examination of the whole spine to determine whether ossified lesions are present in other spinal regions.

The current study is the largest multi-institutional investigation reviewing CT images in the whole spine of patients with cervical OPLL, in which data are reliable with substantial inter-observer agreement. Nevertheless, this study has several limitations. It is based on CT examination of patients with cervical OPLL and is not a population-based study. We did not examine the relationship between clinical symptoms and OPLL lesions. Furthermore, we did not evaluate the OPLL thickness at the thoracolumbar spine, which is often associated with OPLL severity. We must also consider the potential risk of radiation by CT scanning. Most participants in this study underwent CT as a preoperative examination to determine the vertebral level at which the surgical procedure should be performed. Despite these limitations, this study produced important epidemiological data regarding patients with cervical OPLL, which is beneficial for the management of patients with OPLL.

## Conclusion

The incidence of thoracolumbar OPLL was as high as 56.2% in patients with cervical OPLL. The prevalence of thoracolumbar OPLL in these patients increased with the number of levels at which OPLL was present in the cervical spine. Multiple regression analysis revealed that the extent of OPLL in the whole spine was significantly associated with the cervical OP-index, female sex, and obesity.

## Supporting Information

S1 Table Patients’ background and radiologic data sheet. (XLSX)
